# Retreat for Intemperate Women

**Published:** 1863-12

**Authors:** 


### RETREAT FOR INTEMPERATE WOMEN.

The subject of the following communication is one the importance of which can hardly be exaggerated. The unfortunate victims of the vice to which it refers are among the most pitiable objects to which our professional sympathies are ever directed. The experience of nearly every physician must have furnished him with cases of this kind of the most embarrassing character. Household restraints and home influence are little better than worthless in these cases, and the prospect of an asylum where they can be received and tenderly cared for will bring an indescribable relief to many. We have no means of knowing how extensive a provision is required for the purpose in our own State, but we hail the commencement of this movement with the greatest satisfaction, and hope it may meet with the signal success which it deserves.

Retreat for Intemperate Women.—The necessity of making some special provision for the victims of intemperance, partly for the benefit of the individual and partly for that of the community, is beginning to attract general attention, and the subject in its various bearings has been brought before the Massachusetts State Board of Commissioners on Insanity, as among the matters deserving their serious consideration.

Aside from the question of establishing a public asylum for inebriates, the advantages of which would be more naturally confined to the middle and lower classes, it appears that there is yet in New England no place of refuge for intemperate women of good social position except the public and private lunatic asylums, which are unfitted, in the almost unanimous opinion of their superintendents, for the reception of such cases; at many asylums, indeed, admittance being refused to them, alike in justice to the other patients and to the inebriates themselves. The number of applications at the New York General Asylum at Binghamton far exceeds the possible capacity of the building, while the Washingtonian Home in Boston, whose influence for good is already so extended, is for men alone.

In accordance with this apparent want, arrangements have been made by which there will be afforded to a limited number of self-indulgent women, whether addicted to opiates or stimulants, the necessary elements for their cure; namely, voluntary seclusion from temptation, the strictest privacy if desired, a location in the immediate vicinity of the city, and yet unrivalled for purity of atmosphere and beauty of scenery The house selected for the purpose is one constructed with especial reference to a comfortable residence during the winter; attendants will be provided of unexceptionable character, and but few patients will at present be received. For further information application may be made to the Secretary of the Commission, Dr. H. R. Storer, at Hotel Pelham, Boston; the other members of the Board being Hon. Josiah Quincy, Jr., of Boston, and Dr. Alfred Hitchcock, of the Governor's Council, of Fitchburg. It may be stated that the step now taken has the cordial approval and endorsement of His Excellency Governor Andrew, Judge Hoar of the Supreme Court, Drs. James Jackson,Jacob Bigelow, John Jeffries, H. I. Bowditch, J. Mason Warren, Tyler of the Asylum at Somerville, Jarvis of Dorchester, and others of our more prominent citizens.—*Boston Medical and Surgical Journal.*

